# Unveiling the Mary Wollstonecraft’s Self: A Psychoanalytic Reading of “Mary: A Fiction”

**DOI:** 10.1192/j.eurpsy.2025.2408

**Published:** 2025-08-26

**Authors:** I. Hacisalihoglu Aydin, M. Tomsen

**Affiliations:** 1 Mood Disorders Research Program, Depression Center Department of Psychiatry and Behavioral Sciences, University of Louisville School of Medicine, Louisville, United States

## Abstract

**Introduction:**

We can learn a great deal about the power of writing to vanquish the worst forms of psychological trauma by looking closely at Mary Wollstonecraft in the second half of the eighteenth century. Mary Wollstonecraft wrote a Vindication of the Rights of Woman (1792), which is considered by many to be the most important work in feminist thought. Her literary impact went far beyond this foundational work. With the publication of Wollstonecraft’s first semi-fictional autobiography, *Mary: A Fiction* (1787), we can see how her personal life did not follow conventional rules either. Her dedication to individual freedom and social progress was not limited to women. Her own personal life history significantly shaped her influential stance on women’s equality. She was a pioneering advocate for women’s equal treatment to men.

**Objectives:**

This presentation aims to explore how ‘the author,’ Mary, coped with her own life traumas by going beyond the era of her time. By examining her real-life reflections of her fictional characteristics through the lens of psychoanalytic formulations, we seek to gain deeper insights into her inner self and coping mechanisms.

**Methods:**

We critically analyzed *Mary: A Fiction* from a psychoanalytical and literary perspective. By connecting each real-life trauma with its fictional retelling, we uncover Mary’s liberation.

**Results:**

Self-analysis refers to Freud’s exploration and examination of his own psyche, emotions, thoughts, and behaviors in order to gain insight into the unconscious processes that influenced his behavior. In *Mary: A Fiction*, Mary Wollstonecraft wrote her autobiography as a way of her ‘self-analysis’ in order to erase her family’s repression of her ego. In this work, she explores a solitary and challenging self-analytical journey to address the erasure of her identity enforced by her family’s adherence to her mother’s martyrdom to the role of women. Through this process, she constructs a narrative of anger and righteousness to replace the previously “unspeakable” narrative of submission. In his *Memoirs of Mary Wollstonecraft* , Mary’s husband, William Godwin, locates the sadomasochism of her parents’, Elizabeth and Edward John’s patriarchal marriage, as foundational. In response to this trauma, Mary kills off her brother, mother, and father in the first seven chapters of *Mary: A Fiction.* Once her family is erased, so is their erasure of her. Mary can cast off her guilt and give herself the tools to “work through” the traumas of : her tyrannical, abusive, favored brother; the blows of her alcoholic father, and her family’s adherence to her mother’s martyrdom. These were the traumatic cornerstones for Mary’s pain and repressed rage.

**Conclusions:**

Writing a fictional biography based on one’s own life story is a remarkable example of how unresolved conflicts in real life can be reprocessed and lead to eventual healing, similar to the work done in psychoanalysis.

**Disclosure of Interest:**

None Declared

